# Singles’ Reasons for Being Single: Empirical Evidence From an Evolutionary Perspective

**DOI:** 10.3389/fpsyg.2020.00746

**Published:** 2020-05-06

**Authors:** Menelaos Apostolou, Jiaqing O, Gianluca Esposito

**Affiliations:** ^1^Department of Social Sciences, University of Nicosia, Nicosia, Cyprus; ^2^Department of Psychology, Aberystwyth University, Aberystwyth, United Kingdom; ^3^Department of Psychology and Cognitive Science, University of Trento, Trento, Italy; ^4^Department of Psychology, School of Social Sciences, Nanyang Technological University, Singapore, Singapore; ^5^Lee Kong Chian School of Medicine, Nanyang Technological University, Singapore, Singapore

**Keywords:** singlehood, reasons for being single, evolutionary mismatch, mating, mate choice

## Abstract

A relatively large number of people in Western societies are single; that is, they are not involved in any romantic relationship. In this study, we have attempted to investigate the reasons for singlehood by asking singles themselves. A final sample of 648 American singles (307 of them women) rated 92 possible reasons for singlehood. These reasons were classified into 18 broad factors and four general domains. Among the most important reasons were poor flirting skills, freedom, fear of getting hurt, having different priorities, and being too picky. Significant sex and age effects were found across different factors and domains. More specifically, men were more likely than women to indicate that they were single in order to be free to flirt around, and because they were not into family making; while women were more likely to indicate that they were single in order to avoid getting hurt, and because they have considered themselves not to be desirable as mates. Younger people were more likely to indicate that they were single because they had poor flirting skills, because they did not see themselves as desirable mates, and because they did not like commitment; whereas older people were more likely to indicate that they were single in order to be free to do what they have wanted. Findings were examined and discussed using evolutionary theories relating to mate selection and evolutionary mismatch.

## Introduction

In contemporary post-industrial societies, a substantial proportion of adult individuals are single, i.e., they are not involved in any romantic relationship. For instance, a 2005 study found that 32.7% of the adult population in the United States were not in an intimate relationship (Pew Research Center, 2006). This study was replicated in 2013, with the number of people who were not involved in a romantic relationship reported to constitute 35% of the population (Pew Research Center, 2013). Similarly, another study involving a nationally representative sample of American adults has also found that about one in four participants did not have an intimate partner (Rosenfeld et al., 2015). Indeed, singlehood appears to be on the rise. For instance, while 28% of the adult population in the United States were single in 1970, this number has risen to more than 40% in 2002 (DePaulo and Morris, 2005).

Recent theoretical and empirical work has attempted to identify the reasons that have led people to be single (Apostolou, 2015, 2017, 2019; Pepping and MacDonald, 2018), with one study in particular (Apostolou, 2017) offering a list comprising of 76 such reasons. However, none of the subsequent research following that work has investigated the validity of these reasons by focusing exclusively on those who are actually single – which would conceivably provide some much-needed findings straight from the horses’ mouths. Hence, the current study has sought to expand on previous work on this topic by asking singles themselves about their reasons for being single.

As evidenced by the plethora of reasons that have shown to underlie singlehood, which is a complex phenomenon that does not have one causal explanation, the goal of the present manuscript is to identify the factor structure of singles’ reasons for being single, and to explore whether these reasons are associated with singles’ sex and age. Although the nature of this research is largely exploratory, theories from evolutionary psychology can shed light on some patterns that may emerge. In the remainder of this introduction, we will highlight theories and clarify some predictions that may flow from each. In particular, we will contend that, at certain stages in their lives, it may be beneficial for people to be single. We will also argue that the transition from a context in which mate choice was regulated to a context in which it is freely exercised is too brief from an evolutionary perspective for selection forces to adequately adjust psychological adaptations – hence, this might have played a part in individuals facing more difficulties in attracting partners in the present day. We will also explore the evolutionary reasoning behind the notion that people are likely to face issues, such as health problems, which could prevent them from finding a partner. These theories are believed to be complementary, as they could potentially explain different aspects of the singlehood phenomenon.

## The Nature of Singlehood

Humans are sexually reproducing species, which means that, in order to reproduce, individuals need to gain access to the reproductive capacity of the opposite sex. Moreover, children require considerable parental investment until they have reached sexual maturity and are able to reproduce on their own (Clutton-Brock, 1991). In addition, having a partner who provides material and non-material support can greatly increase the survival chances of one’s progeny. These factors have favored the evolution of psychological mechanisms such as romantic love and the cognitive need for intimacy that motivate people to seek partners and to establish long-term relationships so that children can be successfully conceived and nurtured (Buss, 1987, 2017; Symons, 1979). These mechanisms are, in general, evolutionarily effective, as the great majority of people eventually marry and have children (Miller, 2011). Nonetheless, this argument does not explain why so many people remain single for prolonged periods of their lives in the present day. Three main factors have been proposed to account for this phenomenon (Apostolou, 2015, 2017), and will be examined next.

### The Fitness Benefits of Being Single

While it might seem like a paradox on the face of it, not having an intimate partner under certain circumstances could likely increase one’s reproductive success. More specifically, when people search for long-term mates, they are likely to look for traits such as having a good job, high social status and good education that could indicate a high capacity to provide resources, which are required for raising a family (Buss and Schmitt, 1993, 2019; Buss, 2017). Yet, many of these qualities are not innate; for instance, people are not born with a good job. As such, it might pay off for some individuals to allocate their limited resources in developing these qualities prior to their search for long term partners. Such a strategy may not be appropriate for people who are looking for casual partners, and are thus less concerned about their mate value; but it may work well for people who are looking for a long-term partner, and are thus, more concerned about the latter’s mate value. For instance, some people may choose to focus their energy in advancing their careers prior to looking for a marriage partner. Once they have done so, they might then have better chances of mating success, and could therefore divert their resources in attracting suitable long-term partners. Simply put, not having a good job or not having a job at all is a major constraint in the long-term mating market, especially for men; by the same token, having a good job is a major advantage. Accordingly, in terms of success in the long-term mating market, it would be more beneficial for men who have just graduated from college to focus their attention on securing a good job than in securing a long-term mate. Based on this perspective, we would expect singles to indicate reasons such as lacking time for a relationship and career advancement for their current singlehood status.

Furthermore, prospective mates vary considerably in their mate value – for instance, some are younger, better looking, healthier, more intelligent, and have a higher capacity to generate resources than others. Some theories suggest that, it is optimal for mate-seekers to find mates with mate value similar to their own. If they strive for mates of a greater to their own mate value, they might not be able to keep them for long; while if they strive for mates of a lower mate value, they would forgo the benefits of a higher value mate who they could otherwise have attracted (Buss, 2017). Indeed, people tended to prefer as mates individuals who share similar characteristics with them (Hitsch et al., 2010; Watson et al., 2014), which could in turn lead to assortative mating (Watson et al., 2004; Luo, 2017). Nevertheless, such an undertaking is time-consuming, and so it might be beneficial for individuals not to settle down with the first willing mate coming their way, but to stay single until they have found an available mate with a mate value more similar to their own. From this perspective, we would expect singles to indicate being picky and waiting for the right one as reasons for being currently single.

It can also be profitable for mate-seekers to refine their mating skills and acquire some mating experience by engaging in different casual relationships prior to committing to one. By having acquired more experience and by having improved their flirting skills (for more on flirting skills see Apostolou and Christoforou, 2020), people may stand a better chance of attracting a long-term mate of a higher mate value. According to this perspective, we would expect singles to indicate the ability to flirt around with many different people, for instance, as a reason for their singlehood status.

In a similar vein, following the termination of a long-term intimate relationship, it might be beneficial for people to spend some time on their own to reflect on the reasons as to why the relationship has ended, and to improve themselves before reentering the long-term mating market. In addition, as discussed above, children in our species require considerable parental investment in order to stand a good chance in reaching sexual maturity. Thus, it would be useful for men to establish long-term intimate relationships in which children could be adequately supported by their parents. Nevertheless, because men are not constrained by pregnancy, their reproductive success is proportional to the number of women they can gain access to Buss and Schmitt (1993). That is, after sexual intercourse with one partner that has resulted in conception, men still have the potential to inseminate a different one, within a few hours or even minutes. Accordingly, it would be evolutionarily beneficial at times for men to adopt a short-term mating strategy and to seek casual sex with different women instead of committing to a long-term intimate relationship (Buss and Schmitt, 1993, 2019). As such, it is contended that, in certain stages in their lives, it might be beneficial for people to stay single at least for a while.

### The Mismatch Problem

Adaptations are mechanisms which have evolved to interact with specific aspects of the environment in order to produce fitness-increasing outcomes (Irons, 1998; Tooby and Cosmides, 2005). When these aspects change, adaptations may not be equally effective or may become totally ineffective in producing fitness-increasing outcomes. Nonetheless, evolutionary selection forces are exercised on genes, which code for these adaptations, removing genetic variants or alleles, which are not optimal for the novel conditions, and keeping the ones which are. In this regard, these adaptations would eventually adjust to the new environment, so that they interact with it in a fitness-increasing fashion. Yet, this process takes time, and in the interim, there would be several individuals who would suffer fitness penalties because they have adaptations, which are not properly adjusted to the kind of environment which they currently occupy. This is known as the mismatch problem (Crawford, 1998; Maner and Kenrick, 2010; Li et al., 2017), and it has been hypothesized to be one of the reasons behind the observed high prevalence rates of singlehood in post-industrial societies (Apostolou, 2015). In particular, psychological adaptations that are geared toward reliably solving mating and reproductive problems in the ancestral context, might not have been equally effective in doing so in the modern environment, the reason being that the nature of the mating market between the two different time periods is very dissimilar.

More specifically, anthropological and historical evidence has indicated that the selection of a partner was generally regulated in the ancestral context. In addition, evidence from pre-industrial societies, which greatly resembled the way of life of ancestral ones, has shown that the typical avenue for long-term mating was through arranged marriage, where parents chose spouses for their children (Apostolou, 2007, 2010; Walker et al., 2011). Free mate choice had never been the norm in any of the known historical societies, as marriages were typically arranged (Coontz, 2006; Apostolou, 2012). Moreover, men have usually formed male coalitions in order to fight other men and to monopolize their resources and women by force (Tooby and Cosmides, 1988; Ghiglieri, 1999). Anthropological evidence has also indicated that such fights are commonly found in contemporary hunter-gatherer, as well as in agropastoral societies (Chagnon, 1992; Ember and Ember, 1992), with such incidents noted to be more frequent in the latter (Ember and Ember, 1997; Nolan, 2003). This evidence, along with historical and archeological data, suggests that such fights were similarly common in ancestral societies (Keegan, 2004; Bowles, 2009; Puts, 2016), but are considerably less common in modern post-industrial ones (Pinker, 2011). We need to note, however, that forming male coalitions is probably not men’s primary strategy, as mating takes place predominantly in times of peace across societies (Apostolou, 2014).

In Western post-industrial societies, individuals are often free to make their own choice of a mate, and mates are typically not chosen by parents or forced upon individuals by male coalitions. Nevertheless, the transition from a context in which mate choice was largely regulated to a context in which it is freely exercised, has taken place very recently in evolutionary terms, for psychological mechanisms to have enough time to adapt to the new conditions. Thus, people’s adaptations involved in mating have primarily evolved in a context in which mate choice was regulated and male-male competition was strong, and may not work equally well in a context in which mate choice is freely exercised. Therefore, several people may face difficulties in attracting and retaining mates and may consequently end up being single. In this respect, we expect that several people in the present day are single because they have a poor flirting capacity, as this capacity was not necessary in the ancestral context, in which mate choice was regulated. A note of caution though: proposing that individuals might be single as a result of evolutionary mismatch is not the same as suggesting that people are aware of the actual underlying reasons as to why they are single. The reasons people might adopt, and the items proposed in this study, are mainly proximate explanations of an assortment of underlying ultimate causes (e.g., those relating to evolutionary mismatch and sexual selection explanations).

### Constraints

People are likely to face several constraints that prevent them from participating effectively in the mating market. People could develop a serious illness, which might then render them undesirable as mates, and deprive them of the necessary resources required for attracting and keeping mates. Similarly, some individuals may have young children from previous relationships, and would need to allocate considerable resources such as time and money in raising these children, leaving limited resources available for mating effort. In such scenarios, people may lack the necessary resources for participating effectively in the mating market and may have few chances of success if they attempt to do so. Accordingly, they are either forced out of the mating market, or consciously choose to stay away from it, until their constraints have been addressed (Apostolou, 2017). Hence, we expect that some of the reasons for singlehood will cluster in factors and domains that reflect constraints.

### Sex and Age Effects

The reasons for singlehood are unlikely to affect everyone in a similar manner, with sex and age likely to be important predictors. As discussed above, men can benefit more than women from having casual sex with different partners. Consequently, men would be more likely than women to stay single in order to be free to engage in casual relationships. Women, in contrast, allocate more resources to their offspring, and are thus, the scarce reproductive resource which men seek access to (Trivers, 1972). The higher level of parental investment in the form of pregnancy influences women’s choices as they increase their fitness not by having sex with different mates, but by consenting to sex with men who are willing to settle down with them and to provide for them and their children (Buss, 2017). Accordingly, women have evolved to be choosier than men (Buss and Schmitt, 1993, 2019), and their higher level of choosiness may prevent them from being involved in a relationship. Thus, we expect that men will be more likely than women to prefer singlehood in order to be able to have casual sex with different partners. On the other hand, women, as opposed to men, are expected to be more likely to be single because they have not found the right partner.

Allocating resources in order to increase social status and resource generating capacity, which are required for successful participation in the mating market, is an evolutionary problem that typically younger individuals are faced with. As such, younger people are more likely than older ones to be single in order to advance their studies or careers. In addition, younger people are more likely than older ones to stay single in order to refine their flirting skills. Furthermore, older people are more susceptible than younger ones to illnesses and might have offspring that they need to care for. Accordingly, older individuals are more likely than younger ones to face constraints that prevent them from participating effectively in the mating market. In sum, the reasons for singlehood are expected to vary between sexes and between age groups.

### Why Singles Are Single?

To summarize, three main reasons have been proposed to explain why individuals who are single are single: (1) because under certain circumstances being single can increase fitness; (2) because of the evolutionary mismatch between ancestral and modern conditions; and (3) because of different constraints, some people may be less effective in participating in the mating market. Consistent with these arguments, three domains, namely “Difficulties with relationships,” “Freedom of choice,” and “Constraints,” have been identified by previous research (Apostolou, 2017), and they are in line with the three reasons that were discussed above. Apostolou (2017) has also found that men were more likely than women to indicate that they were single in order to be free to flirt around and to do what they have wanted, and because they did not like commitment. On the other hand, women were more likely than men to indicate that they were single because they have had bad experiences from previous relationships, and that they were afraid of change (Apostolou, 2017).

Furthermore, it was also reported that older people were more likely to indicate that they were single because they have had bad experiences from previous relationships, and that they have had issues such as poor health that has kept them back; whereas younger people were more likely to indicate that they were single in order to flirt around, and because they had different life-priorities (Apostolou, 2017). However, one major issue with that study, which the current study is designed to address, is that it asked participants for reasons, which they believed could drive them to be single and not the actual reasons why single people were single – in fact, most of the participants in the sample were either married or were in a relationship – an issue that raises important questions of validity. Although Apostolou (2017) has attempted to produce a list of such reasons using qualitative research methods, subsequent qualitative research has indicated that the list was not comprehensive enough and that it did not include several important reasons for singlehood (Apostolou, 2019). Likewise, Apostolou’s (2017) study was based on the Greek cultural context, and thus, additional research is required to examine reasons for singlehood in different cultural contexts. Although one other qualitative study has also examined 13,429 responses from a Reddit thread asking members for the reasons men were single (Apostolou, 2019), the responses were mainly analyzed conceptually because the qualitative nature of the data did not allow any quantitative statistical analysis to be performed. In addition, Apostolou’s (2019) study was limited to men, so its findings cannot be readily generalized to women.

To our knowledge, these are the only two empirical papers to date, which have attempted to examine the reasons for singlehood, and findings from both have been constrained by important limitations. The current study attempts to contribute to the literature by addressing these limitations and expanding on these research efforts. Accordingly, the current study, through the adoption of findings from previous research, aims to construct a more comprehensive list that would enable us to perform a more accurate taxonomy of the reasons for singlehood and to assess their relative importance.

The current study also aims to address the issue of validity by being the first empirical study to assess an updated list of reasons for singlehood that was based on Apostolou’s (2017) study, using a sample of participants who were actually single. People who do not have a partner could be broadly divided into those who are between relationships, those who are single because they wish to be so, and those who are single because they face difficulties in attracting a partner (Apostolou et al., 2019). Our study aimed to examine the reasons which led to singlehood across singles and not in specific categories of singlehood. Accordingly, we did not differentiate between singles, and we included in our sample all participants who did not have a partner.

Taken together, we asked single people to rate a comprehensive list of reasons as to why they were single. Our theoretical framework makes specific predictions about the different reasons for singlehood. Specifically, we predict that these reasons would cluster in several domains, with one reflecting the fitness benefits for being single, another reflecting the mismatch problem, and another which highlights the kinds of constraints people have been under. Nonetheless, given the complexity of the phenomenon and the plethora of proposed reasons, our study is largely explorative and so factors and domains not predicted by our theoretical framework are likely to be relevant as well. We will examine the relevance of these reasons with people who are single, and we will conduct a principal components analysis in order to assess the importance of the identified factors in this sample. Subsequently, we will also examine the ways in which these factors are influenced by sex and age.

## Materials and Methods

### Participants

Following the ethical approval by a psychology department of a United Kingdom university (ethics approval code: 9322), participants were recruited using a hired agent via the online Amazon Mechanical Turk platform (MTurk)^1^. Informed consent was obtained by all participants. Participants were provided with a nominal financial payment for their involvement. The criteria for participating in the study were as follows: The MTurk workers’ contributions had to be approved minimally for 99% of the time on the online marketplace, and they had to have completed more than 1,000 tasks on the crowdsourcing website beforehand; they also had to be single and have not been involved in any form of romantic relationship at that time of the study; to have been based in the United States and to have been at least 18 years old.

In total, 659 individuals took part in the study initially. Eight of them did not complete the study and so were excluded from the final analyses. In addition, one participant indicated India as his country of residence, while another two reported their marital status as “Other” and “In a relationship” respectively, and hence, they were likewise not used for the final analyses. One male participant did not report his age properly and so was not included in the calculations for the mean age for males or in the factor analyses assessing age differences. The final sample of 647 participants (307 females, 340 males), excluding this participant, has a mean age of 42.2 for women (*SD* = 13.8, Range = 64) and a mean age of 37.2 for the remaining group of men (*SD* = 11.4, Range = 58). Of the whole sample of 648 participants (including the participant with the insufficient age information), 572 of them reported being single, while 76 of them “divorced” (which we assume for the purposes of this study to represent a type of singlehood as well).

### Materials

The study was conducted online and consisted of two sections. Participants were asked to rate several reasons for their singlehood in the initial section, using a five-point Likert scale (1 – Strongly disagree, 5 – Strongly agree). The order of presentation was randomized across participants. In the second part, demographic characteristics were collected (sex, age, and marital status). In order to measure the reasons why people were single, we employed an extended version of the instrument developed by previous research (Apostolou, 2017). The original instrument consisted of 76 reasons, which were identified by using a combination of qualitative and quantitative research methods. We have also added 16 additional reasons, which have been identified by a recent study (Apostolou, 2019), giving us a total of 92 reasons, listed in Table 1.

**TABLE 1:** Classification of the reasons for staying single in factors and domains.

Domains	Factor loadings	Factor loadings
*Factors*	first	second
Reasons	order	order
**Low capacity for courtship**		
*I am not good at flirting*		0.895
I am shy	0.807	
I am very introverted	0.795	
I am terrible at picking up on signals	0.738	
I am not good in flirting	0.708	
I am socially awkward	0.708	
I get high anxiety around women/men	0.581	
I do not know how to start a relationship	0.496	
I do not feel confident	0.465	
I fear rejection	0.428	
I am a boring individual	0.418	
I do not make any effort or make any moves to attract a potential partner	0.336	
I am not good in relationships	0.332	
I have no avenues for meeting available men/women	0.286	
*I am not a desirable mate*		0.862
I am not good looking	0.700	
Because of my weight	0.684	
I have not achieved much in life and I do not think I am attractive as a mate	0.547	
I believe that nobody wants to be with me	0.531	
My financial situation prevents me from having a relationship	0.486	
I fear that my negative aspects will be revealed	0.319	
*I experience sexual difficulties*		0.627
I am not doing very well in the sexual domain	–0.744	
Sometimes I face sexual difficulties	–0.741	
*Commitment scares me*		0.615
Commitment scares me	–0.657	
Love scares me	–0.542	
Change scares me	–0.534	
I do not like commitment	–0.349	
I have not accumulated enough experiences to commit to a relationship	–0.332	
*I have a health/disability problem*		0.614
I have a disability	0.917	
I have a serious health issue	0.898	
I have psychological problems	0.376	
I am going through a period of intense stress and anxiety	0.303	
**Freedom**		
*I want to be free to do whatever I want*		0.897
I want to be able to go wherever I want without needing to answer to anyone	0.831	
I do not want to lose my freedom	0.812	
I want to not have to answer to anyone about what I am doing	0.783	
I do not tolerate restrictions	0.750	
I want to be able to be myself	0.654	
I want to be free to chase my own goals	0.640	
I like to have my own space	0.598	
I want to not feel under pressure	0.596	
I am not willing to make compromises and concessions	0.578	
I want to have fewer obligations	0.526	
I want to avoid the responsibilities that a relationship entails	0.514	
I want to have more choices	0.501	
I want to be able to dress the way I want without having to answer to anyone	0.465	
I want to avoid conflict	0.424	
I prefer to be alone	0.424	
I feel that I need some time alone	0.376	
I got used to be alone	0.276	
*I am not the family type*		0.842
I do not want to have a family	0.846	
I am not the family type	0.822	
I believe that being in a relationship will not make me happier than I am right now	0.377	
I do not feel the emotional need to start a relationship	0.368	
*I have different priorities*		0.712
I want to focus on my career	0.778	
I do not have enough time to devote to a relationship	0.641	
I worry that a relationship is going to be damaging for my career	0.620	
I do not feel ready to start a relationship	0.438	
I have different priorities	0.390	
I believe that I am too old to start a relationship	–0.280	
*I want to spend more time with my friends*		0.695
I want to have more time to spend with my friends	0.565	
I do not want to be alienated from my friends	0.547	
I am doing well right now	0.331	
*I want to be free to flirt around*		0.646
I want to be able to have many casual relationships	0.854	
I want to have a freer sexual life	0.809	
I want to be free to flirt with whoever I want	0.760	
I want to be able to go out more often	0.493	
I want to not get bored	0.351	
**Constraints from previous relationships**		
*I want to devote my attention to my children*		0.666
I have children from a previous relationship	0.887	
I want to devote my attention to my children	0.875	
*I have not gotten over my previous relationship*		0.656
I recently broke up	0.848	
I have not gotten over my previous relationship	0.771	
I am grieving	0.533	
*I fear I will get hurt*		0.557
I am afraid that I will get hurt again	0.740	
I am afraid that my partner will cheat on me	0.718	
I am afraid that my partner will stop loving me	0.692	
I am afraid that the relationship will fail	0.681	
I am afraid that what I will give to the relationship will be wasted	0.663	
I am afraid that I will be disappointed	0.635	
I do not trust men/women	0.595	
I do not trust easily	0.546	
Bad experiences from previous relationships	0.544	
I would not have to worry about where my partner is and what he/she is doing	0.464	
I want to avoid jealousy	0.463	
I am attracted to the wrong men/women	0.348	
I had many failures and I have given up trying	0.334	
*I am too picky*		0.500
I cannot find someone interesting	0.837	
I cannot find the right one	0.758	
I am very picky	0.670	
**Personal constraints**		
*Addictions*		0.616
Because of my addictions (alcohol, drugs etc.)	0.770	
*Sexual orientation*		0.591
Because of my sexual orientation	–0.830	
I need some time to decide about my sexual orientation	–0.828	
My relationship may not be socially acceptable	–0.538	
*I cannot have children*		0.450
I cannot have children	0.738	
*I move often*		0.294
I move often so it is not easy to keep a relationship	–0.750	

## Results

### Factor Structure

We began our analysis by attempting to classify the 92 reasons into broader factors. For this purpose, we employed a principal components analysis using the direct oblimin as the rotation method. The KMO statistic indicated that our sample was very good for a principal components analysis to be performed (KMO = 0.94). Based on the Kaiser criterion (Eigenvalue > 1), 18 factors have been extracted, and are presented in Table 1. The internal consistency of these factors (alpha) ranged from 0.61 to 0.95 with a mean of 78.1. In order to classify these factors into broader domains, a second-order principal components analysis was performed. In particular, 18 new variables were created, which reflected the mean of each extracted factor. Subsequently, a principal components analysis was performed on these variables using direct oblimin as the rotation method. Using the Kaiser criterion (eigenvalue > 1), four domains have been extracted and are presented in Table 1.

### Occurrence

#### Factors

In order to estimate which factor was more likely to lead people to be single, we estimated the means of each one and placed them in a hierarchical order, starting with the one with the highest mean. Note that, no item in each factor was reverse scored, and higher means indicated a higher agreement that the factor in question was responsible for leading participants to be single. The analysis demonstrated that factors such as “I am too picky,” “I want to be free to do whatever I want,” and “I am not good at flirting,” were the most common reported reasons (see Table 2). Using a similar procedure, the four domains were also placed in a hierarchical order. As seen from Table 3, at the top of the hierarchy was “Freedom,” followed by “Low capacity for courtship.”

**TABLE 2:** Sex and age effects for the 18 extracted factors.

		Overall	Women	Men			Age*	
							
Factors	Rank	Mean (*SD*)	Mean (*SD*)	Mean (*SD*)	*p-*value	$\eta_{\text{p}}^{2}$	*p-*value	$\eta_{\text{p}}^{2}$
I am too picky	1	3.17 (1.05)	3.38 (1.05)	2.99 (1.01)	<0.001	0.034	0.665	0.002
I want to be free to do whatever I want	2	2.85 (1.02)	2.86 (1.04)	2.84 (0.99)	<0.001	0.086	< 0.001(+)	0.125
I am not good at flirting	3	2.83 (1.05)	2.76 (1.01)	2.91 (1.08)	<0.001	0.077	< 0.001(−)	0.145
I am not the family type	4	2.57 (1.09)	2.50 (1.06)	2.64 (1.11)	<0.001	0.046	0.023	0.018
I am not a desirable mate	5	2.50 (1.07)	2.42 (1.11)	2.58 (1.03)	<0.001	0.134	< 0.001(−)	0.074
I have different priorities	6	2.42 (0.77)	2.41 (0.76)	2.43 (0.79)	0.273	0.012	< 0.001(−)**	0.233
I fear I will get hurt	7	2.41 (0.92)	2.52 (0.95)	2.32 (0.88)	<0.001	0.092	< 0.001***	0.086
Commitment scares me	8	2.21 (0.93)	2.19 (0.96)	2.26 (0.90)	<0.001	0.062	< 0.001(−)	0.105
I want to spend more time with my friends	9	1.96 (0.79)	1.92 (0.75)	1.99 (0.83)	0.003	0.021	0.002(−)	0.023
I have a health/disability problem	10	1.90 (0.90)	1.88 (0.91)	1.91 (0.90)	0.683	0.004	< 0.001(−)	0.076
I experience sexual difficulties	11	1.83 (1.02)	1.77 (0.98)	1.87 (1.05)	0.089	0.008	0.002(−)	0.019
I want to be free to flirt around	12	1.74 (0.82)	1.56 (0.69)	1.90 (0.88)	<0.001	0.051	0.096	0.014
I have not gotten over my previous relationship	13	1.51 (0.77)	1.56 (0.81)	1.47 (0.72)	0.075	0.011	0.046	0.012
I move often	14	1.42 (0.89)	1.36 (0.83)	1.47 (0.93)	0.266	0.002	0.055	0.006
I want to devote my attention to my children	15	1.38 (0.84)	1.52 (1.03)	1.26 (0.61)	0.002	0.019	0.241	0.004
Sexual orientation	16	1.28 (0.55)	1.30 (0.58)	1.27 (0.53)	0.016	0.016	0.006	0.019
Addictions	17	1.28 (0.74)	1.15 (0.56)	1.39 (0.82)	<0.001	0.020	0.078	0.005
I cannot have children	18	1.21 (0.59)	1.20 (0.60)	1.22 (0.60)	0.574	0.000	0.425	0.001

*The sign of the coefficient of age was placed in parenthesis.**The sign was positive for the “I believe that I am too old to start a relationship” reason.***Age was significant with a negative coefficient for the “I am single because I am afraid that my partner will stop loving me” and significant with a positive coefficient for the “I am single because of bad experiences from previous relationships.”

**TABLE 3:** Sex and age effects for the four extracted domains.

		Overall	Women	Men			Age*	
							
Domains	Rank	Mean (*SD*)	Mean (*SD*)	Mean (*SD*)	*p-*value	$\eta_{\text{p}}^{2}$	*p-*value	$\eta_{\text{p}}^{2}$
Freedom	1	2.31 (0.70)	2.25 (0.67)	2.36 (0.72)	<0.001	0.058	<0.001 (−)	0.066
Low capacity for courtship	2	2.25 (0.77)	2.20 (0.77)	2.30 (0.77)	0.868	0.003	<0.001 (−)	0.071
Constraints from previous relationships	3	2.12 (0.58)	2.24 (0.62)	2.01 (0.51)	<0.001	0.049	0.513	0.005
Personal constraints	4	1.30 (0.41)	1.25 (0.37)	1.34 (0.44)	0.001	0.028	0.004 (−)	0.023

*The sign of the coefficient of age was placed in parenthesis.

Furthermore, we calculated the number of participants who gave scores of “4” or “5” in each of the reasons that composed each of the 18 factors. In this way, we could calculate how many participants were not single due to a particular factor – they gave scores lower than “4” in all the reasons that constitute that specific factor – and how many were single due to that factor – they gave scores of “4” or more to at least one reason that constitutes that specific factor. From Table 4 we can see that the most important factor was the “I am not good at flirting,” followed by the “I want to be free to do whatever I want,” and the “I fear I will get hurt” factor.

**TABLE 4:** The occurrence of factors and domains in the sample.

Factors	Total	Women	Men
I am not good at flirting	89%	91.5%	86.8%
I want to be free to do whatever I want	87.3%	89.3%	85.6%
I fear I will get hurt	80.2%	83.1%	77.7%
I have different priorities	79.5%	81.4%	77.7%
I am too picky	75.2%	82.1%	68.9%
I am not a desirable mate	64.8%	61.2%	68%
I am not the family type	61.4%	62.2%	60.7%
Commitment scares me	55.1%	55%	55.1%
I have a health/disability problem	43.5%	45.6%	41.6%
I want to spend more time with my friends	34.7%	36.5%	33.1%
I want to be free to flirt around	23.5%	18.9%	27.6%
I experience sexual difficulties	18.4%	17.9%	18.8%
I have not gotten over my previous relationship	18.1%	22.1%	14.4%
I want to devote my attention to my children	9.3%	15%	4.1%
Sexual orientation	7.9%	8.8%	7%
I move often	5.1%	4.9%	5.3%
I cannot have children	5.1%	4.9%	5.3%
Addictions	3.2%	2%	4.4%
**Domains**			
Low capacity for courtship	94.6%	94.5%	94.7%
Freedom	92.7%	92.8%	92.7%
Constraints from previous relationships	92.1%	93.5%	90.9%
Personal constraints	16%	16.3%	15.8%

*For factors, the percentages refer to the number of participants who indicated a score of “4” or “5” in any of the reasons composing each factor. For domains, the percentages refer to the number of participants who indicated a score of “4” or “5” in any of the reasons composing each factor which loaded to each domain.*

#### Domains

We performed a similar analysis for the four domains. We calculated how many of the participants indicated that they were single due to at least one factor composing the domain. For instance, if a participant gave a score of “4” or “5” in any of the reasons composing the “I am not good at flirting” factor, we considered that they were single due to this factor, and due to the domain “Low capacity for courtship,” which this factor was a constitute of. As we can see from Table 4, the most prevalent domain in our sample was “Low capacity for courtship” followed by “Freedom.”

### Comorbidity

In order to examine whether participants were single only due to reasons loaded in one domain, or for reasons spread in different domains, we estimated how many participants indicated that they were single in more than one domain (i.e., they indicated at least one factor that composed the domain as important, see above). We found that 5.6% of all participants indicated all four domains as important in explaining why they were single. To put it differently, this finding suggested that all four domains contributed to why 5.6% of the participants were single. We repeated the analysis by dropping the “Personal constraints” domain, which had the lower prevalence rate in the sample. We found that 17.1% of the participants indicated all three domains as important. Finally, we repeated the analysis only for the “Low capacity for courtship” and the “Freedom” domains, which were the most prevalent in our sample, and we found that 23.5% indicated that both domains contributed to their singlehood.

### Significant Sex and Age Effects

In order to identify significant effects of sex and age for each factor, we performed a series of MANCOVAs, where the dependent variables were the reasons composing each factor, and the independent variables were sex and age. MANCOVA is a statistical test that allows the examination of the effect of a combination of independent variables that are continuous and categorical on a dependent factor, which consists of more than one variable (Field, 2018). Our analysis has identified 18 different factors, each consisting of several reasons. Thus, the use of such a test is appropriate for investigating the effect of sex and age on each factor. In cases in which the factor was composed of only one reason, ANCOVA was performed instead. Overall, 18 tests were performed, and in order to avoid the possibility of alpha inflation, we applied Bonferroni correction in which the alpha was set to 0.003 (0.05/18). Accordingly, any effect with a *p*-value of more than 0.003 should not be considered significant.

The analyses indicated significant sex and age effects for most factors (Table 2; see also **Supplementary Table A** in the **Supplementary Material**). As indicated by the effect sizes, the largest sex-difference was in relation to the “I am not a desirable mate” factor. Women indicated that they were more likely than men to be single because of their weight, while men indicated that they were more likely than women to be single because they had not achieved much in life, and thus, they were not desirable as mates, and because their financial situation prevented them from being in a relationship. The second highest sex-difference was for the “I fear I will get hurt” factor, where women scored significantly higher than men. The third in magnitude was the “I want to be free to do what I want” factor. The total means were very similar, but there were significant sex differences in this dimension, where reasons such as having fewer obligations and avoiding the responsibilities of a relationship were rated significantly higher by men than women. Men also gave higher scores than women to the “I am not the family type,” the “Commitment scares me,” and the “I want to be free to flirt around” factors. A substantial sex-difference was also found for the “I am not good at flirting” factor, where men indicated more than women that they were single because they experienced anxiety when they were close to an opposite-sex partner, because they considered themselves boring, and because they were not good in relationships. Women, on the other hand, were more likely than men to indicate that they did not have avenues for meeting available mates.

As indicated by the effect size, the largest age effect was for the “I have different priorities” factor, where younger individuals gave higher scores than older ones, as originally predicted. The second largest effect was for the “I am not good at flirting” factor, where younger individual also gave higher scores than older ones. In addition, a large age effect was found for the “I want to be free to do what I want” factor, where older individuals gave higher scores than younger ones, as originally predicted.

Finally, we applied a series of MANCOVAs in order to estimate significant sex and age effects on each of the four domains. In particular, the factors were entered as the dependent variables, and participants’ sex and age were entered as the independent variables. The results are presented in Table 3, where we can see that significant sex and age effects were found for most domains. As indicated by the effect size, the largest sex-difference was in the “Freedom” domain, while the largest effect of age was in the “Low capacity for courtship” domain.

## Discussion

Our analyses indicate that the 92 potential reasons for singlehood in our current sample could be classified into 18 broad factors, with the most common ones being poor flirting skills, willingness to be free, fear of getting hurt, having different priorities, and being too picky. The 18 factors could be grouped further into four general domains, with the highest rated ones being ‘Freedom’ and ‘Low capacity for courtship.” Significant sex and age effects were found across different factors and domains. For instance, men were more likely than women to indicate that they were single in order to be free to flirt around, and because they were not into family making; while women were more likely than men to indicate that they were single in order to avoid getting hurt, and because they were not perceiving themselves to be desirable as mates. Additionally, younger people were more likely to indicate that they were single because they had poor flirting skills, they were not desirable as mates, and because they did not like commitment; whereas older people were more likely to indicate that they were single in order to be freer to do what they have wanted.

More specifically, in relation to the “Low capacity for courtship” domain, people have indicated that they were single because they were not good at flirting; for instance, they were shy, introverted, unable to pick up signals of interest, and they lacked confidence. These difficulties in flirting could be explained by the mismatch between ancestral and modern conditions: in an ancestral context, where marriages were arranged and mating was forced, flirting skills had a limited effect on the capacity to attract a mate. Regardless of whether people were introverted or had a poor capacity to pick up clues relating to mating interest, it would have made little difference given that most marriages were arranged by parents in that prehistoric context. Accordingly, selection forces had been weak in shaping good flirting capacity, which is necessary, however, in a modern context where choice is freely exercised. The drastic change in environmental conditions, from mate choice being regulated or forced to one where it is freely exercised, coupled with the evolutionary recency of this change – marriages were arranged only a few generations ago in most Western societies (Coontz, 2006) – can explain why poor flirting skills were the most frequent reason for being single, with nearly 90% of the participants indicating that this was one of the reasons why they did not have intimate partners.

People who scored highly in this domain also appeared to consider themselves to be undesirable as mates, predominantly due to their looks. The evolutionary mismatch problem is also likely to be at play here. When arranging a marriage, parents have little interest in the looks of their prospective in-laws (Apostolou, 2014), and this trait has played little role in predicting success in fights and wars as well. Accordingly, selection forces acting on traits, which are considered attractive to mates and/or mechanisms regulating attention to looks were likely relatively weak, therefore resulting in several individuals possessing a physical appearance that might not have been widely acknowledged as attractive in the modern context. People ascribed much more importance to the looks of a mate than that of an in-law (Perilloux et al., 2011; Apostolou, 2014), and such differential preferences would likely suggest that one’s looks are much more important in a context of free mate choice in predicting mating success. As a consequence, several people who may not have an attractive appearance will likely experience difficulties in their pursuits.

The evolutionary mismatches in other aspects of the environment may have also affected mating success. More specifically, recent technological developments have made food readily available to nearly everyone in Western societies. Food-intake regulation mechanisms have not had time to adjust to these conditions, and thus they still operate as if food is in short supply, resulting in many people becoming obese (Breslin, 2013). In turn, being overweight might cause difficulties in participating effectively in the mating market, which likely explains why many of our participants have indicated that their weight was a reason for being single. Similarly, people in contemporary societies are largely overly exposed to TV images, movies and images on the Internet of people who are significantly above average in terms of looks and significantly below average in terms of weight (Eyal and Te’eni-Harari, 2013; Boothroyd et al., 2016). However, TV, cinema and the Internet are evolutionary novel and as such, people have not yet evolved to remain rooted in reality based on information from these sources. In effect, many mate-seekers, by having their standards for looks and weight determined by information from the media, may start to feel that they are overweight or ugly, thereby demotivating them to look for mates.

Within the “low capacity for courtship” domain, health and disability problems are also prominent issues. Indeed, we expected the factor that represents these issues to load in the “constraints” domain and not in here. One possible reason is that people may have considered a health problem or a disability as something that could compromise their capacity to be effective in the mating market. Further research and empirical validation will enable us to better understand the processes at play here.

Moving on, people might also prefer to be single in order to be free to do the things they have wanted (e.g., flirting around, advancing their careers, and enhancing their social network by spending more time with friends), as exemplified by the “Freedom” domain in this study. Participants have also indicated possible reasons such as being unwilling to make compromises and to undertake the obligations that a relationship will entail, as well as not being interested in having a family. Within the context of our theoretical framework, this domain is related to the fitness benefits of singlehood. Maintaining an intimate relationship requires the allocation of resources such as time and making compromises such as not having sex with other individuals. People who are single have fewer obligations and experience fewer compromises on their time in order to advance in their careers. Also, by not committing to a relationship, people may be able to have casual relationships with different partners. Doing so could enable them to refine their flirting skills, and to gain more mating experience that would enable them to attract better long-term partners. This strategy could be especially profitable for the evolutionary fitness of men, whose reproductive success is strongly correlated with the number of women they could gain sexual access to. Moreover, by not committing to a relationship and by flirting around, people may get a better sense of the mating market and of their own mate value. This domain, along with the “Low capacity for courtship” one, were the most prevalent in our sample, suggesting that the primary reasons as to why some people are single in the United States context were a consequence of difficulties in attracting mates and a preference to be free from the restrictions of an intimate relationship.

In addition, participants also reported that they were single due to reasons relating to their previous relationships, as indicated by the “Constraints from previous relationships” domain. Factors within this domain included having children and having ongoing feelings for their previous partners. Consistent with these findings, an older study has found that, for women, the presence of children from a prior marriage reduced the likelihood of remarriage (Buckle et al., 1996). One possible reason for these findings is that single people who have children may fear that a prospective partner may harm their own children (see Daly and Wilson, 1988), hence resulting in the preference to stay single. In addition, bad experiences from previous relationships were also reported to prevent people from going into a relationship, as some participants feared that they will get hurt again. The evolutionary mismatch problem is similarly likely to be at play here. Due to evolutionary mismatch, people might not do well in intimate relationships in the modern context and might have accumulated many negative relationship experiences because of that, which in turn could have rendered them less likely to desire engaging in an intimate relationship in the future.

Another reason within this domain is being too picky. This factor can be explained in terms of the fitness benefits of singlehood, as well as the mismatch problem. Prospective partners might vary considerably in their mate value; thus, it could be beneficial for people to avoid entering a relationship with the first available mate, and to stay single until they have found one who matches their own mate value instead. Nevertheless, because parents have played a huge role in controlling mate choice for their children in ancestral societies, there was an absence of strong selection pressures on the latter with regards to refining adaptations involved in mate choice. Because of that, some individuals might be excessively picky in the contemporary context – they may for instance, overestimate their own mate value and attempt to attract mates who may be significantly beyond their reach, thereby resulting in them remaining single.

Furthermore, the current findings suggest that people may also stay single due to reasons associated with “Personal constraints,” such as one suffering from addictions and infertility, having a homosexual orientation, or having to travel often. Being gay or lesbian could prevent people from being in a relationship because same-sex attraction is associated with a strong social stigma (Fone, 2000); thus, people may prefer to remain reticent about their sexual orientation and be single than to enter in a same-sex or opposite-sex union. Another reason is that, people may live in small cities or villages, where, apart from the constraints of discrimination against such sexual preferences, there are also significantly fewer same-sex options (Apostolou et al., 2018). This domain was demonstrated to be one of the least prevalent ones, however, perhaps because prevalence rates of one not being able to have children, having a serious addiction, and being homosexual, respectively, are relatively low in the population.

Sex differences are found in several factors. As indicated by the effect sizes, the largest one is in relation to the “I am not a desirable mate” factor, where women were much more likely to indicate issues with their weight as compared to men; the opposite is true for issues pertaining to a lack of achievement. This outcome reflects the sex differences in terms of mate preferences: men would typically place more value on the looks of a prospective mate, while women would more likely be concerned about the social standing and wealth of a prospective mate (Buss, 1989, 2017). Accordingly, men and women who do not, or who think that they do not excel in these dimensions are more likely to be demotivated in seeking mates and to stay single as a consequence.

In addition, single men have also assigned higher scores than single women to the “I am not the family type,” “Commitment scares me,” and the “I want to be free to flirt around” factors. These sex differences are likely accounted for by casual sex being more beneficial for the fitness of men than for women (Buss and Schmitt, 2019). A substantial sex difference is also found for the “I am not good at flirting” factor. This difference probably reflects the cultural expectation that men should initiate courtship (Buss, 2017), which in turn suggests that difficulties in doing so would have a higher impact on them than on women. Women, on the other hand, were more likely to report having fewer opportunities to meet available mates. If men are expected to initiate courtship, but face difficulties in doing so (for instance, they may be disinclined from flirting with females as a result of their perceived inability), women may, as a consequence, feel that they do not have enough mate options to choose from.

With respect to age, the largest difference was in the “I have different priorities” factor, where younger individuals assigned higher scores than older ones. Instead of directing their resources in finding and keeping a partner, younger people might potentially be investing their time in building qualities such as having a good job instead – attributes, which will enable them to attract individuals with a higher mate value in the future. Older people have most likely already done so in terms of job achievements for instance, which could possibly explain the observed age effect. In addition, younger participants were also more likely to indicate that the “I am not good at flirting” factor was an issue underlying their singlehood than their older counterparts, perhaps because older people tended to have accumulated more relationship experience, and thus have had more opportunities to refine and improve on their flirting skills.

Our findings have revealed several similarities in relation to previous research in this area. The domains we have extracted in this study are similar to those identified by Apostolou (2017). In particular, the “Freedom” domain is very similar to the “Freedom of choice” domain in Apostolou’s (2017) study, while the “Low capacity for courtship” and the “Personal constraints” domains appear to correspond to the “Difficulties with relationships” and the “Constraints” domains in Apostolou’s (2017) study, respectively. In both studies, significant sex differences have been identified, with men regarding freedom as a more important reason for being single than women. There are also differences between the current findings and those of Apostolou’s (2017) study: In the present study, we have extracted one additional domain, namely “Constraints from previous relationships,” which was not found in Apostolou’s (2017) study. In addition, “Low capacity for courtship” was the second most important domain in the present study, while the “Difficulties with relationships” was the most important domain in Apostolou’s (2017) study.

These differences may reflect variations in the study design – the current study has employed a more inclusive list of reasons and the sample was composed only of singles, whereas Apostolou’s (2017) study utilized a shorter list of reasons, involving mostly non-singles. Differences may also simply reflect cultural differences. For instance, American culture is more individualistic than Greek culture, which can likely explain why the “Freedom” domain has a higher mean in the present study. Considerably more studies in different cultural contexts are necessary to examine accurately how cultural factors could affect the reasons underlying singlehood.

The current research is not without limitations, one being that it is based on self-report data. What we have measured here are the reasons that single people think may have led to their singlehood, which may not necessarily be the actual reasons for their singlehood status. While we believe that people in general have a good understanding of why they are single, their understanding is unlikely to be totally accurate. Participants’ responses may have suffered from the problem of introspection: they may have produced an explanation for their behavior, which could be inaccurate because they do not have direct introspective access to their mental processes (Nisbett and Wilson, 1977). Also, because the present study did not measure sexual orientation, and given that homosexuality and bisexuality is found in less than 10% of the population (LeVay, 2010), our sample was most probably predominantly heterosexual. The mating market for people who are either homosexual or bisexual may differ in many respects from the mating market for heterosexual people, which means that the hierarchy of reasons we have found here may be different across different sexual orientation groups. Future studies need to focus specifically on examining the reasons due to which non-heterosexual people are single as well. Furthermore, the study was confined to an American sample, so our findings may not necessarily be generalizable to different cultural settings. In addition, we have attempted to construct an inclusive instrument for measuring the reasons for singlehood; however, the complexity of the phenomenon suggests that there may be additional reasons, which may not have been adequately captured in this study. For instance, safety concerns may lead some people to not delay entering the mating market. Such concerns did not emerge in any of the previous qualitative studies on singlehood, but future studies need to examine whether they do indeed prevent people from forming intimate relationships. Moreover, some of these reasons may be specific to Western cultures, and thus, may not be equally applicable to non-Western cultures. A large scale, cross-cultural research study is perhaps necessary in identifying the different reasons underlying singlehood.

Furthermore, where our sample is concerned, we do not know whether participants have had romantic relationships in the past. There may be some differences between people who have had several relationships in the past and those who only have had a few or none. In addition, we have employed an evolutionary perspective in order to interpret our findings. Yet, other theoretical perspectives may also be used in order to provide further insights for understanding the reasons for singlehood. In the same vein, some of the current findings could be interpreted from a life history perspective, which contends that, among other things, mate choices are an outcome of the compromise every individual has to make in terms of the quantity and quality of one’s progenies, and the amount of investment in these descendants in response to environmental pressures (Lummaa, 2007). Nevertheless, given that the measure we have utilized in relation to the reasons for singlehood was already 92-item long, and that additional questions stemming from a life history perspective would render the instrument to be excessively onerous for participants to answer effectively, such an approach was not adopted in this study. Future research should attempt to examine the phenomenon from a life history angle nonetheless, which could potentially provide additional useful insights.

The current research attempted to shed light regarding the reasons for singlehood in those who were single. Yet, the complexity of the phenomenon requires considerably more work in order to achieve greater understanding of the issue. Future research needs to examine the reasons, which have led people to be single in different cultural contexts, as different cultural conditions could produce different challenges. In addition, future research work needs to identify different factors, which are also likely to predict these reasons and the possible interactions between them. For instance, we expect that having qualities, which are valued in the mating market, such as good looks and wealth, could also interact with sex, rendering men more likely to be single in order to be able to have casual relationships with different partners. Future research may also focus on employing the current findings in developing appropriate interventions, which could enable men and women to be more successful in their mating efforts. For instance, the results of the current research suggest that a common reason for singlehood is the lack of good flirting skills. Accordingly, interventions could be developed with the aim to enhance people’s flirting capacity.

In sum, the current findings have offered a comprehensive list of reasons as to why single individuals are single, as reported by singles themselves. These reasons were explicated through a consideration of evolutionary factors, such as the notion that singlehood may partly have been an outcome of the large discrepancy in terms of environmental conditions between the ancestral context and the modern world. Considerably more work in this area is necessary, however, if this complex phenomenon is to be fully understood.

## Data Availability Statement

The datasets presented in this article are not readily available in order to preserve the participants’ confidentiality. Requests to access the datasets should be directed to the corresponding author.

## Ethics Statement

This study was carried out in accordance with the recommendations of the American Psychological Association with online informed consent from all participants. All participants gave online informed consent in accordance with the Declaration of Helsinki. The protocol was approved by the Aberystwyth University ethics committee (ethics approval code: 9322).

## Author Contributions

MA designed the study, analyzed the data, and wrote the main manuscript text. JO developed the ethics application for the study, assisted in coordinating the data collection process, and has helped with editing the manuscript. GE facilitated the data collection process. All authors reviewed the manuscript.

## Conflict of Interest

The authors declare that the research was conducted in the absence of any commercial or financial relationships that could be construed as a potential conflict of interest.

## Footnotes

1 www.mturk.com
